# Moving-Target Tracking in Airport Airside Operations Using AIMM-STUKF

**DOI:** 10.3390/s26010166

**Published:** 2025-12-26

**Authors:** Jianshu Gao, Yinuo Dang, Yuxuan Zhu, Wenqing Xue

**Affiliations:** 1 School of Transportation Science and Engineering, Civil Aviation University of China, Tianjin 300300, China; 2 School of Electronic Information and Automation, Civil Aviation University of China, Tianjin 300300, China; 2023022245@cauc.edu.cn (Y.D.); 2024022208@cauc.edu.cn (Y.Z.); 2024022240@cauc.edu.cn (W.X.)

**Keywords:** interacting multiple model filtering, strong tracking theory, airport target tracking, AIMM-STUKF, model probability, airport map information

## Abstract

In this paper, we propose a mobile target tracking method for airport movement areas based on an adaptive interacting multiple model framework combined with a strong tracking unscented Kalman filter, referred to as the AIMM-STUKF algorithm. The objective is to enhance real-time tracking accuracy, improve model adaptability, and strengthen robustness against abrupt disturbances in complex airport environments. The proposed AIMM-STUKF adopts a standard STUKF formulation within the overall tracking framework, thereby enhancing responsiveness to maneuvering targets. An exponential correction factor is further constructed based on posterior model probability differences to adaptively adjust the Markov transition matrix, enabling self-adaptive mode switching. In addition, airport map information is incorporated to impose constraints on the position components of the filtered state estimates, enhancing the adaptability of the algorithm to the airport operational environment. Experimental validation is conducted through Monte Carlo simulations using representative trajectories that reflect realistic airport operational characteristics. Comparative results with the standard IMM-UKF and two existing AIMM-UKF algorithms demonstrate that the proposed AIMM-STUKF achieves superior performance in terms of tracking accuracy, model matching consistency, mode-switching responsiveness, and robustness against sudden disturbances.

## 1. Introduction

With the continuous advancement of science and technology, intelligent development has become a primary driving force in the global civil aviation industry [1]. The cooperative operation of automated and manually driven special-purpose vehicles within airport movement areas has emerged as a key trend in modern airport infrastructure development, thereby imposing increasingly stringent requirements on operational safety. Consequently, achieving high-precision, real-time tracking and localization of maneuvering targets in the airside area has become critically important.

As the fundamental component of autonomous driving systems, target tracking and localization are required to ensure continuity, real-time capability, and high accuracy. However, maneuvering targets within airport movement areas are characterized by large spatial coverage, highly diverse motion states, and significant prediction uncertainty. Under such conditions, single-model tracking algorithms exhibit inherent limitations and are unable to satisfy the demands of real-world operations [2,3]. The Interacting Multiple Model (IMM) approach, originally proposed for Systems with Markovian switching coefficients [4], provides an effective solution and has since been widely applied in various complex scenarios, including exo-atmospheric ballistic tracking, high-maneuver fighter tracking, near-space hypersonic target tracking, underwater vehicles, and airborne surveillance systems [5,6,7,8,9,10]. In modern target tracking research, maneuver modeling and model switching are widely regarded as effective means for improving tracking performance in complex motion scenarios. Reference [11] proposes a Bayesian framework for inferring dynamically evolving group leadership structures from noisy observations, by developing a destination-driven leader–follower model and employing sequential Monte Carlo methods to jointly enhance leadership identification and tracking accuracy. Reference [12] proposes a Bayesian framework based on α-stable Lévy state-space models for maneuvering object tracking and intent prediction, achieving noticeable performance improvements over conventional Gaussian dynamic models.

The optimization of IMM-based algorithms in existing research can be broadly classified into three categories: model-set selection, filtering algorithm enhancement, and transition probability adaptation. Reference [13] adopts the Current Statistical (CS) model and exploits its advantages to enhance the robustness of maneuvering-target tracking under mixed line-of-sight and non-line-of-sight conditions. Reference [14] employs a model set comprising the Constant Velocity (CV) model, a three-dimensional fixed-center Constant Speed and Constant Turn Rate (CSCTR) model with adaptive turn-rate adjustment, and the CS model. Reference [15] introduces an improved CS-Jerk model as one of the sub-models, thereby increasing the consistency between the assumed motion models and the true target dynamics. Reference [16] utilizes an autoregressive (AR) predictive model to characterize the motion state and updates the motion equations online, enabling a more accurate description of target dynamics and improving overall algorithmic robustness. Reference [17] designs an IMM set based on the Clohessy–Wiltshire (C–W) equation, an augmented C–W model, and a fading-memory C–W model, effectively accommodating various maneuvering modes of space targets and improving the matching fidelity to the real system with relatively low computational complexity. Reference [18] models vehicle dynamics using the Constant Velocity–Coordinated Turn (CV–CT), Constant Velocity–Constant Acceleration (CV–CA), and Constant Acceleration–Coordinated Turn (CA–CT) motion models. However, these model-selection strategies are generally more suitable for open-space or highly maneuvering targets rather than the constrained airport surface environment. This limitation arises from an application-scenario mismatch, as airport operations must strictly adhere to specific safety regulations and operational constraints.

Extended Kalman filtering (EKF) and unscented Kalman filtering (UKF) are widely used for state estimation in highly nonlinear systems [19]. However, EKF relies on the accurate computation of Jacobian matrices and therefore exhibits insufficient robustness when the system dynamics are strongly nonlinear or subject to model uncertainties. In contrast, UKF employs the Unscented Transformation (UT) [20,21,22,23,24], which propagates a carefully selected set of sigma points through nonlinear functions to more effectively capture the mean and covariance evolution. As a result, UKF typically achieves superior filtering performance in nonlinear estimation tasks. To further improve filtering performance, several enhanced approaches have been proposed. In reference [25], an improved adaptive Sage–Husa algorithm is integrated with UKF to ensure the positive definiteness of the estimated measurement-noise covariance; however, its effectiveness in mitigating prediction errors during model-switching transients and at the onset of target maneuvers remains limited. Reference [26] introduces a dimension-mismatched interacting IMM-UKF framework that groups multiple motion models into two composite filters, enabling parallel three-dimensional filtering; nevertheless, the non-equal-dimensional state mapping may introduce additional numerical uncertainty in practical implementations. In reference [27], adaptive UKF and robust UKF are fused by probabilistically weighting their estimation outputs, thereby enhancing the adaptability and robustness of the overall filter; however, the parallel operation of two filters leads to a significant increase in computational complexity.

Transition-probability adaptation represents an effective pathway for enhancing IMM-based algorithms. Reference [28] proposes an end-to-end learning-based adaptive IMM algorithm, in which a neural network estimates the transition probability matrices (TPMs) in real time. A generalized recurrent neural network is trained directly on tracking tasks using an end-to-end strategy, reducing dependence on large datasets and improving model generalization; however, its performance remains highly dependent on the availability and quality of training data. In refs. [29,30,31], adaptive adjustment strategies based on the definition of error compression ratios or compression ratio ratios are proposed, in which posterior information is exploited to correct the prior model information. Specifically, reference [30] overcomes the limitation in reference [29] that only two models can interact by redefining the error-compression ratio to support three-model interaction; however, the tracking error increases when the system does not undergo mode switching. In reference [31], the compression-ratio definition is further refined, and the adaptive adjustment conditions originally applicable only to two-model cases are extended to multi-model scenarios. Nevertheless, compression-ratio-based approaches are highly sensitive to measurement noise and transient variations, which may result in spike errors during model-switching instants. Reference [32] proposes a measurement-driven adaptive correction strategy for model transition probabilities, which enhances the influence of the matched model while suppressing that of the mismatched ones, yet exhibits reduced tracking accuracy during model transitions. However, when the target undergoes significant maneuvers, relatively large tracking errors are observed in both position and velocity estimates. In refs. [33,34,35], model transition probabilities are adaptively modified based on model likelihood values. Specifically, Reference [34] further refines the correction parameters proposed in [33], effectively mitigating singularity issues in the transition matrix. Building upon reference [34], reference [35] introduces an additional correction step using a decision window, enabling a two-stage refinement of the Markov transition matrix and more effective exploitation of posterior information. Nevertheless, likelihood-based correction approaches inherently rely on the numerical stability of the likelihood function values, which may lead to difficulties in regulating correction strength and insufficient steady-state stability of the model probabilities.

Airport surface target tracking exhibits pronounced application-specific characteristics. Target motion is subject to strict spatial constraints imposed by runways, taxiways, and aprons; maneuvering behaviors are relatively limited, whereas motion-state transitions occur frequently. Moreover, this scenario is safety-critical, imposing higher requirements on the stability and feasibility of tracking results. Consequently, methods that rely solely on historical statistical information or complex probability correction mechanisms struggle to simultaneously achieve rapid responsiveness and steady-state stability in such environments. Although the aforementioned algorithms improve model probability accuracy and overall tracking performance, their position and velocity root-mean-square errors (RMSEs) remain insufficient to meet the high-precision requirements of complex airport operational environments. In addition, reliance on historical model information in these approaches tends to slow down model switching, leading to response lag during motion-state transitions.

To address the aforementioned practical engineering challenges, this study proposes an application-oriented AIMM-STUKF target tracking framework that jointly considers model-switching responsiveness, tracking accuracy, and operational suitability for airport scenarios. The proposed framework is characterized by system-level integration of a standard STUKF formulation within an adaptive interacting multiple model architecture, in conjunction with an adaptive model-probability correction mechanism and airport map–based constraints. The standard STUKF is embedded into the overall framework to adaptively balance the weighting between historical information and current measurements. Meanwhile, unlike approaches that rely on error-compression ratios or likelihood-function-based tuning, the Markov transition probability matrix is adaptively and exponentially adjusted according to posterior model-probability differences. In addition, airport map information is incorporated to impose constraints on the position components of the filtered state estimates, thereby further improving tracking accuracy and ensuring that the estimation results remain consistent with airport-surface operational requirements.

## 2. System Tracking Model Establishment

The state model and measurement model describe the dynamic behavior of the system. Specifically, the system input is characterized by the state equation, which consists of a deterministic time function and stochastic process noise, whereas the system output is described by the measurement equation, which is a function of the state vector and is corrupted by measurement noise. For a target governed by multiple motion models, the corresponding state-transition equation is formulated as shown in Equation (1):

(1) $$X_{k}=FX_{k-1}+w_{k-1}$$

The measurement equation is given as Equation (2):

(2) $$Z_{k}=H\left(X_{k}\right)+v_{k}$$

In Equations (1) and (2), k denotes the sampling instant; $X_{k}$ represents the state vector, which contains the true state information of the target at time k; The state transition matrix at time k is denoted by F; $w_{k-1}$ denotes the process noise, which reflects the environmental disturbances affecting the motion of the moving target, with covariance $Q_{k}$; $Z_{k}$ is the measurement vector, representing the observed range and bearing of the target at time k; $v_{k}$ denotes the measurement noise, which is related to the monitoring equipment and sensors, with covariance $R_{k}$; $w_{k-1}$ and $v_{k}$ are mutually uncorrelated two-dimensional zero-mean Gaussian white noise processes; $\delta_{kj}$ is the Kronecker delta function, leading to Equation (3):

(3) $$\begin{array}{l} E\left[w_{k}\right]=0,\;\mathrm{cov}\left[w_{k},w_{j}\right]=Q_{k}\delta_{kj},\\ E\left[v_{k}\right]=0,\;\mathrm{cov}\left[v_{k},v_{j}\right]=R_{k}\delta_{kj},\\ \mathrm{cov}\left[w_{k},v_{j}\right]=0 \end{array}$$

In the IMM-UKF framework, the construction of the model set must comprise multiple motion models that collectively characterize all possible system behaviors, and its formulation must comply with the Bayesian principles of completeness and independence [36]. Moreover, the tracking accuracy of the algorithm is closely related to the target’s motion model—the more consistent the assumed motion model is with the actual motion of the target, the higher the tracking accuracy achieved.

The motion states of moving targets in airport movement areas are generally characterized by three types of motion: uniform linear motion and uniformly accelerated motion on taxiways and runways, as well as coordinated turn motion at intersections between taxiways or between taxiways and runways. These three motion states are, respectively, described by the constant velocity (CV) model, the constant acceleration (CA) model, and the coordinated turn (CT) model.

The motion characteristics of the moving target are represented by its position coordinates x and y, velocity components $\overset{\cdot }{x}$ and $\overset{\cdot }{y}$, acceleration components $\overset{\cdot \cdot }{x}$ and $\overset{\cdot \cdot }{y}$, and the angular velocity ω during turning. At a given sampling instant k, the motion state under the constant velocity model is expressed by Equation (4); the motion state under the constant acceleration model is defined by Equation (5); and the motion state under the coordinated turn model is represented by Equation (6).

(4) $$X_{k}={\left(x_{k},y_{k},{\dot{x}}_{k},{\dot{y}}_{k}\right)}^{T}$$

(5) $$X_{k}={\left(x_{k},y_{k},{\dot{x}}_{k},{\dot{y}}_{k},{\overset{..}{x}}_{k},{\overset{..}{y}}_{k}\right)}^{T}$$

(6) $$X_{k}={\left(x_{k},y_{k},{\dot{x}}_{k},{\dot{y}}_{k},\omega \right)}^{T}$$

## 3. IMM-UKF Algorithm

### 3.1. UKF Algorithm

Initialization:

(7) $$\left\{\begin{array}{l} {\hat{X}}_{0|0}=E\left[X_{0}\right]\\ P_{0|0}=E\left[\left(X_{0}-{\hat{X}}_{0|0}\right){\left(X_{0}-{\hat{X}}_{0|0}\right)}^{T}\right] \end{array}\right.$$

The sigma point set is calculated as shown in Equation (8), where a total of 2n+1 sampling points are generated:

(8) $$\left\{\begin{array}{l} \eta_{k|k-1}^{i}={\hat{X}}_{k|k-1},\;i=0\\ \eta_{k|k-1}^{i}={\hat{X}}_{k|k-1}+\left[\sqrt{\left(n+\lambda \right)P_{k|k-1}}\right],\;i=1,\ldots ,n\\ \eta_{k|k-1}^{i}={\hat{X}}_{k|k-1}-\left[\sqrt{\left(n+\lambda \right)P_{k|k-1}}\right],\;i=n+1,\ldots ,2n \end{array}\right.$$

Calculation of the corresponding weights of the sigma points:

(9) $$\left\{\begin{array}{l} W_{m}^{0}=\frac{\lambda }{n+\lambda }\\ W_{c}^{0}=\frac{\lambda }{n+\lambda }+\left(1+\beta -\alpha^{2}\right)\\ W_{m}^{i}=W_{c}^{i}=\frac{\lambda }{2\left(n+\lambda \right)},\;i\neq 0 \end{array}\right.$$

In Equations (8) and (9), λ denotes the scaling parameter, defined as $\lambda =\alpha^{2}\left(n+\kappa \right)-n$, which is used to reduce the overall prediction error; α is a scaling factor, typically chosen as a small positive number; and β is the secondary scaling factor, selected as a non-negative number.

The one-step predicted values of the sigma points are then computed. Based on these predictions, the system state estimate and the predicted state covariance are obtained:

(10) $$\delta_{k|k-1}^{i}=f\left(\eta_{k-1|k-1}^{i}\right)$$

(11) $${\hat{X}}_{k|k-1}=\sum_{i=0}^{2n}W_{m}^{i}\delta_{k|k-1}^{i}$$

(12) $$P_{k|k-1}=\sum_{i=0}^{2n}W_{c}^{i}\left(\delta_{k|k-1}^{i}-{\hat{X}}_{k|k-1}\right)\cdot {\left(\delta_{k|k-1}^{i}-{\hat{X}}_{k|k-1}\right)}^{T}+Q_{k-1}$$

Based on the estimated state and covariance, a new set of sigma points is generated using the unscented transformation (UT). These sigma points are then propagated through the measurement equation to obtain the predicted measurement values:

(13) $$\gamma_{k|k-1}^{i}=h\left(\delta_{k|k-1}^{i}\right)$$

(14) $${\hat{Z}}_{k|k-1}=\sum_{i=0}^{2n}W_{m}^{i}\gamma_{k|k-1}^{i}$$

Computation of the predicted mean and covariance:

(15) $$P_{k|k-1}^{zz}=\sum_{i=0}^{2n}W_{c}^{i}\left(\gamma_{k|k-1}^{i}-{\hat{Z}}_{k|k-1}\right)\cdot {\left(\gamma_{k|k-1}^{i}-{\hat{Z}}_{k|k-1}\right)}^{T}+R_{k}$$

(16) $$P_{k|k-1}^{xz}=\sum_{i=0}^{2n}W_{c}^{i}\left(\eta_{k|k-1}^{i}-{\hat{X}}_{k|k-1}^{i}\right)\cdot {\left(\gamma_{k|k-1}^{i}-{\hat{Z}}_{k|k-1}\right)}^{T}$$

The Kalman gain calculation, system state update, and covariance update are given by the following equations:

(17) $$K_{k}=P_{k|k-1}^{xz}{\left(P_{k|k-1}^{zz}\right)}^{-1}$$

(18) $${\hat{X}}_{k|k}={\overset{\wedge }{X}}_{k|k-1}+K_{k}\left(Z_{k}-{\hat{Z}}_{k|k-1}\right)$$

(19) $$P_{k|k}=P_{k|k-1}-K_{k}P_{k|k-1}^{zz}K_{k}^{T}$$

### 3.2. IMM-UKF Algorithm

The standard IMM-UKF algorithm consists of four main components: model interaction, filtering, model probability update, and state estimate fusion.

In the model interaction step, the conditional initialization and reinitialization are performed for each model, and the interacting inputs for each filter are computed using the model transition probabilities. During the filtering step, each motion model is processed through a UKF, where the interacting inputs and measurement values are used to compute the state estimate at the current time step. Next, the model probabilities are updated based on the filtering results, reflecting the likelihood that each motion model matches the current target dynamics. Finally, in the state estimate fusion step, the individual model estimates are weighted and combined according to the updated model probabilities to produce the overall fused estimate.

Based on Bayesian inference, the recursive process of the IMM-UKF algorithm from time step k−1 to k consists of four steps, as follows:

(1) Model Interaction

The result of each iteration cycle determines the initialization state for the subsequent cycle. The result from the previous time step is used to initialize the current state estimate and its corresponding covariance matrix. Let $\mu_{k-1|k-1}^{ij}$ denote the interaction probability from model i to model j at time k−1; ${\hat{X}}_{k-1|k-1}^{j}$ represent the state estimate; and $P_{k-1|k-1}^{j}$ represent the corresponding covariance estimate, with i,j=1,2,…,r. After interaction computation, the initial conditions for the $j^{th}$ filter input at the current time k step are given by:

(20) $$\begin{array}{c} {\hat{X}}_{k-1|k-1}^{oj}=\sum_{i=1}^{r}{\hat{X}}_{k-1|k-1}^{j}\mu_{k-1|k-1}^{ij}\\ P_{k-1|k-1}^{oj}=\sum_{i=1}^{r}\mu_{k-1|k-1}^{ij}\left[P_{k-1|k-1}^{j}+\left({\hat{X}}_{k-1|k-1}^{j}-{\hat{X}}_{k-1|k-1}^{oj}\right)\cdot {\left({\hat{X}}_{k-1|k-1}^{j}-{\hat{X}}_{k-1|k-1}^{oj}\right)}^{T}\right] \end{array}$$

(2) Filtering

The mixed state estimate and its corresponding covariance at time k, obtained through the interaction computation, are used as the filtering inputs for mode j. Each motion model employs a UKF to perform state estimation based on its respective dynamic characteristics. Consequently, the filtering outputs at time k are the updated state estimate $X_{k|k}^{j}$ and the estimated covariance $P_{k|k}^{j}$.

(3) Model Probability Update

Model probabilities are updated using likelihood functions, where each model’s likelihood represents the degree of consistency between the target motion state and the corresponding motion model.

Assume that, at time k, the innovation $V_{k}^{j}$ and its covariance $S_{k}^{j}$ obtained from the filter follows a Gaussian distribution. Then, the likelihood function $\Lambda_{k}^{j}$ for model j and the updated model probability $\mu_{k}^{j}$ are expressed as follows:

(21) $$\Lambda_{k}^{j}=\frac{1}{\sqrt{|\;2\pi S_{k}^{j}}}\exp\left\{-\frac{1}{2}{\left(V_{k}^{j}\right)}^{T}{\left(S_{k}^{j}\right)}^{-1}V_{k}^{j}\right\}$$

(22) $$\mu_{k}^{j}=\frac{\Lambda_{k}^{j}C^{j}}{\sum_{j=1}^{r}\Lambda_{k}^{j}C^{j}}$$

(4) State Estimation Fusion

The state estimates and covariance matrices obtained from each sub-model filter at time k are combined through weighted summation, where the weight of each model is determined by its updated model probability. Consequently, the final fused state estimate ${\hat{X}}_{k|k}$ and covariance estimate $P_{k|k}$ are given by:

(23) $${\hat{X}}_{k|k}=\sum_{j=1}^{r}\mu_{k}^{j}\cdot {\hat{X}}_{k|k}^{j}$$

(24) $$P_{k|k}=\sum_{j=1}^{r}\mu_{k}^{j}\left[P_{k|k}^{j}+\left({\hat{X}}_{k|k}^{j}-{\hat{X}}_{k|k}\right)\cdot {\left({\hat{X}}_{k|k}^{j}-{\hat{X}}_{k|k}\right)}^{T}\right]$$

## 4. Proposed AIMM-STUKF Algorithm

In practical applications, the Unscented Kalman Filter (UKF) may suffer from enlarged estimation errors, slow convergence, or even divergence. In the standard IMM-UKF framework, the Markov transition matrix is typically predetermined based on prior knowledge or manually specified fixed probabilities, which fails to accurately represent the true switching behavior among the target’s motion models and consequently degrades tracking accuracy and responsiveness. Moreover, maneuvering targets operating within airport environments are subject to highly complex and dynamic conditions; when real-world observations and contextual information are not sufficiently incorporated, the algorithm may suffer from model-matching inconsistencies and degraded localization performance during field deployment.

To address the above issues, this study integrates strong tracking theory with the UKF by employing an STUKF in the filtering stage. A fading factor is introduced to dynamically adjust the Kalman gain, enabling the filter to respond more rapidly to new measurements and enhancing estimation stability under abrupt state variations. Additionally, a correction factor is introduced to modify the model probability transition matrix, enhancing the robustness and sensitivity of the algorithm in complex or dynamic environments. Furthermore, the dynamic information of moving targets obtained from airport surveillance systems is fused with airport map data within the algorithm, improving its adaptability to airport-specific operational conditions.

### 4.1. STUKF Algorithm

The strong tracking filter (STF) is characterized by two sufficient conditions: minimization of the state estimation covariance and mutual orthogonality of the residual sequences at all time steps. These conditions can be expressed as follows:

(25) $$E\left[\left(X_{k}-{\hat{X}}_{k}\right){\left(X_{k}-{\hat{X}}_{k}\right)}^{T}\right]=\min$$

(26) $$E\left[\varepsilon_{k+i}\varepsilon_{k}^{T}\right]=0;\;k=0,1,\ldots ;\;i=1,2,\ldots$$

(27) $$\varepsilon_{k}=Z_{k}-{\hat{Z}}_{k|k-1}$$

When the system state evolves smoothly and the assumed motion model accurately matches the true target dynamics, the filter can satisfy both conditions simultaneously. However, in practical maneuvering target state estimation scenarios, frequent changes in the target motion pattern may lead to model mismatch, making it difficult to maintain the orthogonality of the residual sequence at all time steps. Therefore, a fading factor $\lambda_{k}$ is introduced into the predicted covariance $P_{k|k-1}$ to adaptively adjust the Kalman gain $K_{k}$ in real time, thereby enforcing the orthogonality of the residual sequence.

The computation of the fading factor is given as follows:

(28) $$\lambda_{k}=\max\left(1,\frac{tr\left[N_{K}\right]}{tr\left[M_{K}\right]}\right)$$

(29) $$\left\{\begin{array}{c} N_{k}=V_{k}-{\left(P_{k|k-1}^{xz}\right)}^{T}{\left(P_{k|k-1}\right)}^{-1}Q_{k}{\left(P_{k|k-1}\right)}^{-1}\left(P_{k|k-1}^{xz}\right)-\beta R_{k}\\ M_{k}=P_{k|k-1}^{zz}-{\left(P_{k|k-1}^{xz}\right)}^{T}{\left(P_{k|k-1}\right)}^{-1}Q_{k}{\left(P_{k|k-1}\right)}^{-1}\left(P_{k|k-1}^{xz}\right)-R_{k} \end{array}\right.$$

(30) $$V_{k}=\left\{\begin{array}{l} \varepsilon_{1}\varepsilon_{1}^{T},k=1\\ \left(\rho V_{k-1}+\varepsilon_{k}\varepsilon_{k}^{T}\right){\left(1+\rho \right)}^{-1},k>1 \end{array}\right.$$

In Equations (32)–(34), β denotes the weakening parameter, whose value typically lies within $\left[1,+\infty \right)$. $V_{k}$ represents the innovation covariance matrix, and ρ is the forgetting factor, with its value generally within $\left(0,1\right]$.

The incorporation of the fading factor into the covariance matrix is given as follows:

(31) $$P_{k|k-1}=\lambda_{k}\sum_{i=0}^{2n}W_{c}^{i}\left[Q_{k-1}+\left(\delta_{k|k-1}^{i}-{\hat{X}}_{k|k-1}\right){\left(\delta_{k|k-1}^{i}-{\hat{X}}_{k|k-1}\right)}^{T}\right]$$

Based on the state vector updated after incorporating the fading factor $\lambda_{k}$, the Kalman gain is computed, and the state estimate and covariance matrix are updated as follows:

(32) $$K_{k}=P_{k|k-1}^{xz}{\left(P_{k|k-1}^{zz}\right)}^{-1}$$

(33) $${\hat{X}}_{k|k}={\hat{X}}_{k|k-1}+K_{k}\left(Z_{k}-{\hat{Z}}_{k|k-1}\right)$$

(34) $$P_{k|k}=P_{k|k-1}-K_{k}P_{k|k-1}^{zz}K_{k}^{T}$$

### 4.2. Definition of the Correction Factor

The correction factor is defined as the exponential function of the difference between model probabilities:

(35) $$\chi_{k}^{j}=\exp\left[\mu_{k}^{j}-\mu_{k-1}^{j}\right]$$

The model probability transition matrix is adaptively adjusted based on the correction factor. When the target motion state changes, the probability difference between the matched model and mismatched models increases. The proposed correction factor nonlinearly amplifies this difference, thereby adaptively enhancing the transition probability toward the matched model while suppressing transitions to mismatched models. The resulting rapid convergence of model probabilities effectively shortens the duration during which mismatched models dominate the state fusion process, reduces transient errors caused by model mismatch, and consequently improves tracking accuracy and stability. Let n denote the tuning parameter, with $\left(0\leq n\leq 1\right)$. The corresponding correction procedure is formulated as follows:

(36) $$\left\{\begin{array}{c} p_{*}^{ij}={\left[\frac{\chi_{k}^{j}}{\chi_{k}^{i}}\right]}^{n}\cdot p^{ij},\;i\neq j\\ p_{*}^{ij}={\left[\chi_{k}^{j}\right]}^{n}\cdot p^{ij},\;i=j \end{array}\right.$$

Since the sum of transition probabilities from one model to another at time k must equal 1, i.e., each row of the Markov transition matrix is required to sum to unity, a normalization operation must be applied to the corrected transition matrix. The corresponding normalization procedure is given as follows:

(37) $$p^{ij}=\frac{p_{*}^{ij}}{\sum_{j=1}^{r}p_{*}^{ij}}$$

The magnitude of the diagonal elements of the transition probability matrix directly affects the stability and switching behavior of the model probabilities. Diagonal elements that are too small may cause fluctuations in the model probabilities during steady-state operation, whereas excessively large diagonal elements may suppress the model switching speed and reduce the responsiveness to changes in the true motion state. From the perspective of Markov chain stability, when the transition probability matrix satisfies the diagonal dominance property, the model probabilities are more likely to converge toward and remain stable around the matched model. Therefore, to ensure diagonal dominance of the Markov matrix while enabling adaptive probability adjustment, and to balance model switching sensitivity with steady-state stability, a lower-bound threshold is imposed on the diagonal elements, which is set to th=0.9. If any diagonal element of the normalized matrix falls outside the specified threshold range, it is modified accordingly. The adjustment process is expressed as follows:

(38) $$\begin{array}{l} p_{*}^{ij}=th\\ p_{*}^{ij}=\left(1-th\right)\frac{p^{ij}}{1-p^{ii}} \end{array}$$

### 4.3. Proposed AIMM-STUKF Algorithm

To ensure that the estimated target trajectories conform to the airport operational framework, this study introduces the Airport Map Database (AMDB) as a spatial constraint within the STUKF filtering stage, enabling the correction of state estimates that violate airport operational areas. The utilization of map information is realized through map registration, by which the airport map database is constructed, and the AMDB is subsequently employed to constrain post-filtering position estimates that fall outside the admissible operational domain.

In this work, the CAD map of a selected airport is first simplified, and the key airport surface elements are delineated in accordance with the requirements of the DO-272B standard. These elements are then imported into ArcMap, where map registration is performed using ground control points (GCPs) obtained from in situ GNSS measurements. Upon completion of the registration process, the airport surface operational areas are represented as polygonal regions. Together with their associated semantic information, an AMDB is constructed to describe the admissible operational space as a set of polygons defined within a unified coordinate system.

**Figure 1:**
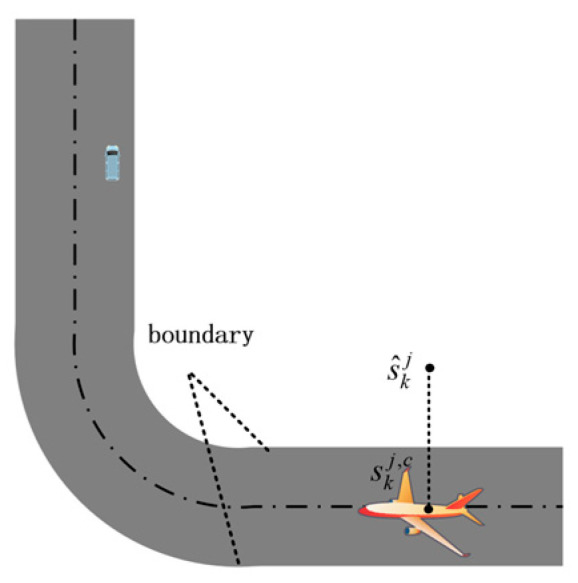
Schematic illustration of position constraint.

The map information is employed to impose constraints on the position components of the post-filtering state estimates, which mainly involves feasibility checking of whether the estimated position lies within the admissible operational domain, as well as position correction, as illustrated in Figure 1.

Let the STUKF-based updated state estimate corresponding to mode j at time k be denoted as ${\hat{X}}_{k|k}^{j}$, and its position component can be expressed as:

(39) $${\hat{s}}_{k}^{j}=C{\hat{X}}_{k|k}^{j}$$

In Equation (39), C represents a position selection matrix that extracts the positional components from the state vector.

The feasible airport surface operational area is represented as the union of multiple polygonal regions, as defined in Equation (40). A standard point-in-polygon (PIP) test is applied to examine the estimated position ${\hat{s}}_{k}^{j}$. Based on the test result, a decision is made as to whether map-constrained position correction should be applied. The detailed correction procedure is given in (41).

(40) $$D=\overset{N}{\underset{n=1}{\cup }}D_{n}$$

(41) $$\left\{\begin{array}{cc} s_{k}^{j,c}={\hat{s}}_{k}^{j} & ,\;{\hat{s}}_{k}^{j}\in D\\ correction & ,\;{\hat{s}}_{k}^{j}\notin D \end{array}\right.$$

A geometric projection approach is adopted to map the deviated position onto the nearest feasible region. The corrected position can be expressed as:

(42) $$s_{k}^{j,c}=\pi \prod_{D}{\hat{s}}_{k}^{j}=\arg\underset{q\in D}{\min}∥q-{\hat{s}}_{k}^{j}{∥}_{2}$$

The corrected position is subsequently incorporated into the state vector, while the remaining state components are kept unchanged, yielding the map-constrained state estimate as follows:

(43) $$\left\{\begin{array}{c} {\hat{X}}_{k|k}^{j,c}={\hat{X}}_{k|k}^{j}+C^{T}d_{k}\\ d_{k}=s_{k}^{j,c}-{\hat{s}}_{k}^{j} \end{array}\right.$$

Since the map-constrained correction constitutes a deterministic geometric operation rather than a stochastic measurement update, the state covariance matrix remains unchanged. The corrected state estimate is subsequently utilized for state fusion and final output, whereas the original STUKF estimate is retained for model probability updating within the IMM framework.

**Figure 2:**
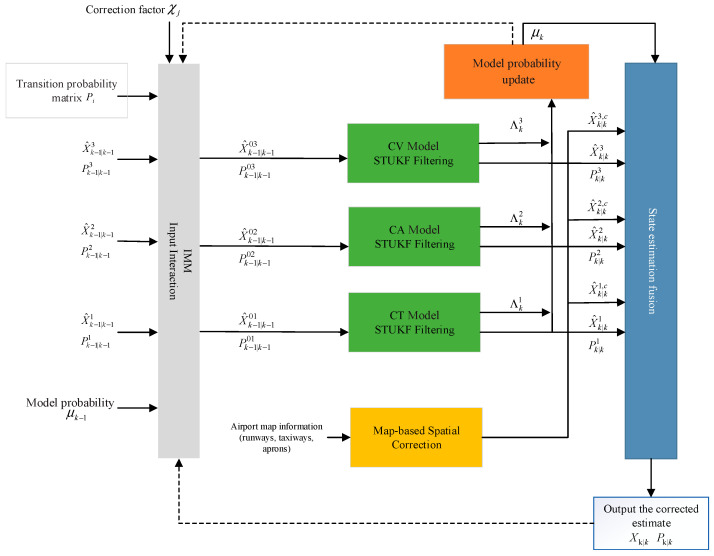
Flowchart of the AIMM-STUKF algorithm.

Based on the exponential of the model probabilities and the airport map information, the flowchart of the corrected AIMM-STUKF algorithm is illustrated in Figure 2. The detailed steps of the algorithm are as follows:

(1) Based on the operational regulations of airports and the fundamental motion characteristics of ground targets in the movement area, a motion model set $M=\left\{m_{1},m_{2},\ldots m_{r}\right\}$ is constructed to represent the system’s possible motion patterns. The selection of specific models is determined according to the target’s motion region and behavior. In the airport movement area, ground vehicles and aircraft primarily exhibit constant acceleration and constant velocity motion along runways and taxiways, as well as constant-turn motion when transitioning through taxiway-to-taxiway or taxiway-to-runway intersections. Therefore, considering the motion characteristics of the maneuvering target, the proposed motion model set in this study consists of the Constant Velocity (CV) model, the Constant Acceleration (CA) model, and the Coordinated Turn (CT) model.
(2) In the interaction step, suppose that the target is associated with model i at time k−1 and with model j at time k. The model probability prediction is updated using the exponential correction factor defined by the model probability subtraction in (35). The corresponding mixing weights are then computed according to (44). Subsequently, the mixed state estimate and mixed covariance obtained from (20) are used as the initial conditions for the filtering step.

(44) $$\mu_{k|k-1}^{j}=\sum_{i=1}^{r}p_{ij}^{\prime }\mu_{k-1}^{i}$$

(45) $$\mu_{k-1}^{ij}=\frac{p_{ij}^{\prime }\mu_{k|k-1}^{j}\mu_{k-1}^{i}}{\mu_{k|k-1}^{i}}$$

(3) In the filtering update stage, each motion model employs its corresponding STUKF filter to perform state estimation. The Kalman gain is computed according to (7)–(19) and (25)–(34), followed by the state and covariance updates under each respective model.
(4) In the map-constrained processing stage, the position component of the post-STUKF state estimate is evaluated according to (39)–(43). Based on the evaluation result, map-based constraints are imposed when necessary. It should be noted that the constrained state estimate is only used for subsequent state fusion and final output, and therefore does not affect the update of the state covariance matrix or model probability computation within the IMM framework.
(5) The likelihood function and the corresponding model probabilities are subsequently derived from the innovation sequence obtained via the STUKF, along with its associated innovation covariance, as expressed in:

(46) $$v_{k}^{j}=Z_{k}-{\hat{Z}}_{k|k-1}^{j}$$

(47) $$\Lambda_{k}^{j}=\frac{1}{\sqrt{|\;2\pi P_{k|k-1}^{zzj}}}\exp\left\{-\frac{1}{2}{\left(v_{k}^{j}\right)}^{T}{\left(P_{k|k-1}^{zzj}\right)}^{-1}v_{k}^{j}\right\}$$

(48) $$u_{k}^{j}=\frac{u_{k|k-1}^{j}\Lambda_{k}^{j}}{\sum_{j=1}^{r}u_{k|k-1}^{i}\Lambda_{k}^{i}}$$

(6) Fused output estimation:

(49) $${\hat{X}}_{k|k}=\sum_{j=1}^{r}\mu_{k}^{j}\cdot {\hat{X}}_{k|k}^{j,c}$$

(50) $$P_{k|k}=\sum_{j=1}^{r}\mu_{k}^{j}\left[P_{k|k}^{j}+\left({\hat{X}}_{k|k}^{j}-{\hat{X}}_{k|k}\right)\cdot {\left({\hat{X}}_{k|k}^{j}-{\hat{X}}_{k|k}\right)}^{T}\right]$$

(7) The state estimates and associated covariance parameters obtained at the current time step are used as the initial inputs for the next time step. This procedure is then repeated iteratively until the end of the tracking cycle.

## 5. Experiments and Results

### 5.1. Experimental Design

(1) Simulation scenario

**Figure 3:**
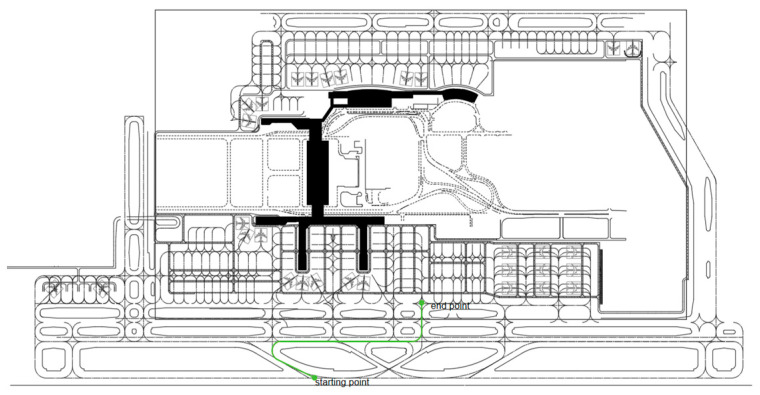
A simplified airport surface map, where the green curve represents the trajectory of the guide vehicle.

**Figure 4:**
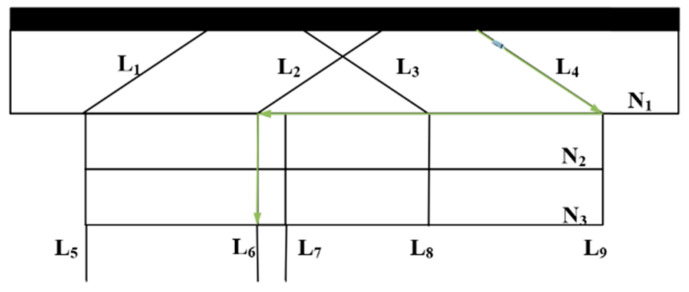
Trajectory of the guidance vehicle, where the green arrow represents the motion direction of the guidance vehicle.

Simulation experiments are conducted based on the actual motion characteristics of airport surface mobile targets. The simulated trajectory of the follow-me vehicle is shown in Figure 3 and Figure 4. The initial position and velocity of the vehicle are set to (800 m, 900 m) and 2 m/s, respectively. The vehicle first accelerates with a constant acceleration of 0.1 m/s^2^ and then moves at a constant velocity along a direction forming an angle of 60° with the *X*-axis for 30 s, corresponding to the CA and CV motion models. Subsequently, it performs a right turn for 6 s with a constant angular velocity, which corresponds to the CT model. The vehicle then continues to travel straight along the *X*-axis at a constant velocity for 60 s under the CV model, followed by a uniform left-turn maneuver modeled by the CT model. Finally, the vehicle changes direction and moves along the *Y*-axis at a constant velocity for 20 s, after which the motion sequence terminates.

Two experimental scenarios are designed to evaluate the tracking performance under different operating conditions. Scenario 1 represents normal driving, where the target follows the predefined trajectory and velocity profile under the influence of nominal process noise only. Scenario 2 introduces abrupt disturbances to assess robustness. Specifically, two impulsive perturbations were injected during the straight-line segment and the turning maneuver, which are the most susceptible phases to sudden dynamic changes. These settings enable a comprehensive examination of the algorithms’ tracking accuracy and recovery capability in both nominal and highly perturbed environments.

(2) Parameter settings

The sampling interval is set to 1 s. The initial model probabilities are uniformly assigned as 1/3. The initial Markov transition probability matrix is $P=\left[0.9,0.05,0.05;0.05,0.9,0.05;0.05,0.05,0.95\right]$. The weakening parameter is set to 1.5. 

A smaller forgetting factor improves responsiveness to maneuver-induced state variations, while potentially increasing estimation variability, whereas a larger value enhances steady-state stability at the expense of reduced responsiveness. Similarly, a lower diagonal dominance threshold in the transition probability matrix increases model-switching sensitivity but may result in oscillatory model probabilities, whereas a higher threshold promotes probability stability while suppressing switching sensitivity. In this study, the forgetting factor and the diagonal dominance threshold are set to 0.98 and 0.9, respectively, to achieve a balanced trade-off between responsiveness and stability.

(3) Comparison methodology

To validate the performance of the proposed AIMM-STUKF algorithm, 100 Monte Carlo simulation runs are conducted under two operating scenarios: normal driving (scenario 1) and driving subjected to external disturbances (scenario 2). The algorithms used for comparison include the standard IMM-UKF (denoted as Method 1), two AIMM-UKF variants (denoted as Method 2 and Method 3, respectively), and the proposed AIMM-STUKF algorithm. The first AIMM-UKF method was introduced in [32] and the second method was proposed in [34].

(4) Performance metrics

The evaluation focuses on two aspects: model matching capability and tracking accuracy. The model matching effectiveness is assessed by comparing the model probability trajectories of different algorithms at various time steps and maneuvering phases. The tracking accuracy is quantified in terms of trajectory estimation performance and the root mean square error (RMSE) of position and velocity, computed as follows:

(51) $$P_{RMSE}=\sqrt{\frac{\sum_{i=1}^{N}{\left(x_{i}^{\prime }-x_{i}\right)}^{2}}{N}}$$

(52) $$V_{RMSE}=\sqrt{\frac{\sum_{i=1}^{N}{\left(v_{i}^{\prime }-v_{i}\right)}^{2}}{N}}$$

### 5.2. Experimental Results

(1) Tracking Results.

By conducting 100 Monte Carlo runs, the position RMSE and velocity RMSE obtained under the normal-driving condition for the four algorithms are summarized in Table 1, while the corresponding RMSE values under the disturbance-injected condition are reported in Table 2.

**Figure 5:**
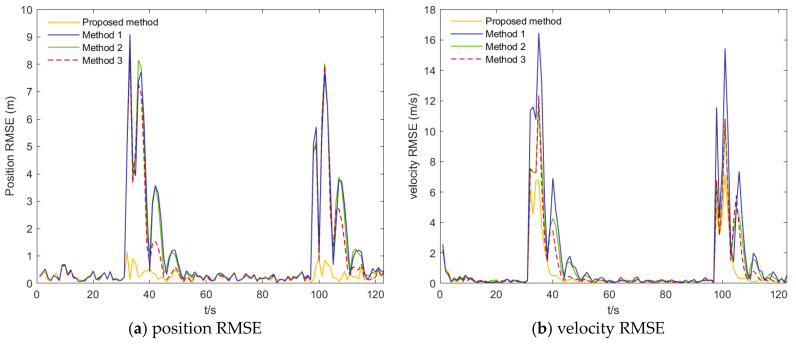
Position RMSEs and Velocity RMSEs of scenario 1.

**Figure 6:**
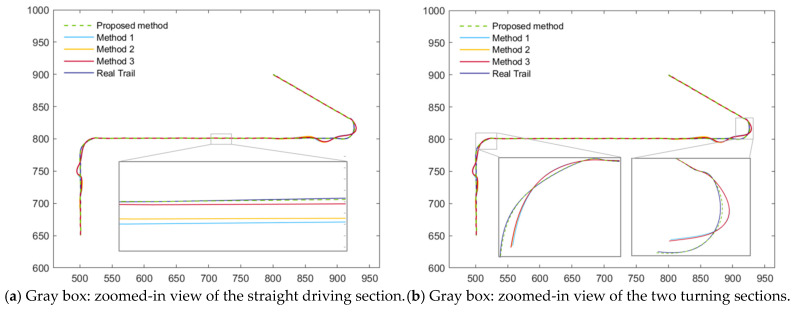
Estimated trajectories of scenario 1.

**Figure 7:**
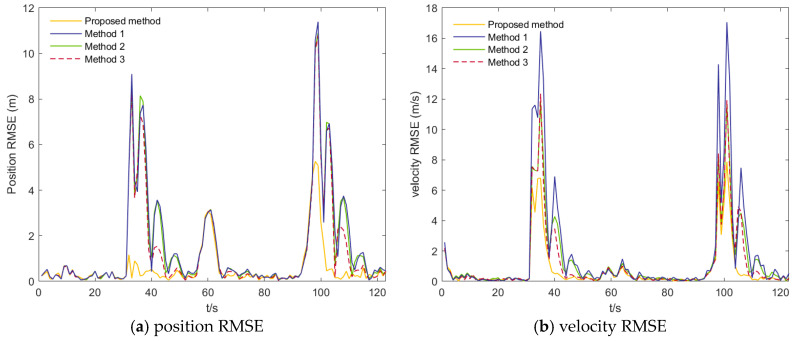
Position RMSEs and Velocity RMSEs of scenario 2.

**Figure 8:**
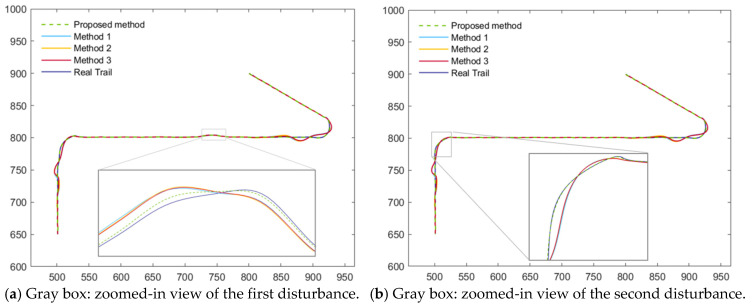
Estimated trajectories of scenario 2.

The tracking performance of the four algorithms under the two scenarios is illustrated in Figure 5, Figure 6, Figure 7 and Figure 8. Figure 5 presents the position and velocity RMSEs of Scenario 1 obtained from a representative Monte Carlo run. Figure 6 shows the estimated trajectories in Scenario 1 along with several locally magnified segments for clearer visualization. Figure 7 displays the position and velocity RMSEs under Scenario 2, while Figure 8 depicts the corresponding estimated trajectories and their locally enlarged views.

**Figure 9:**
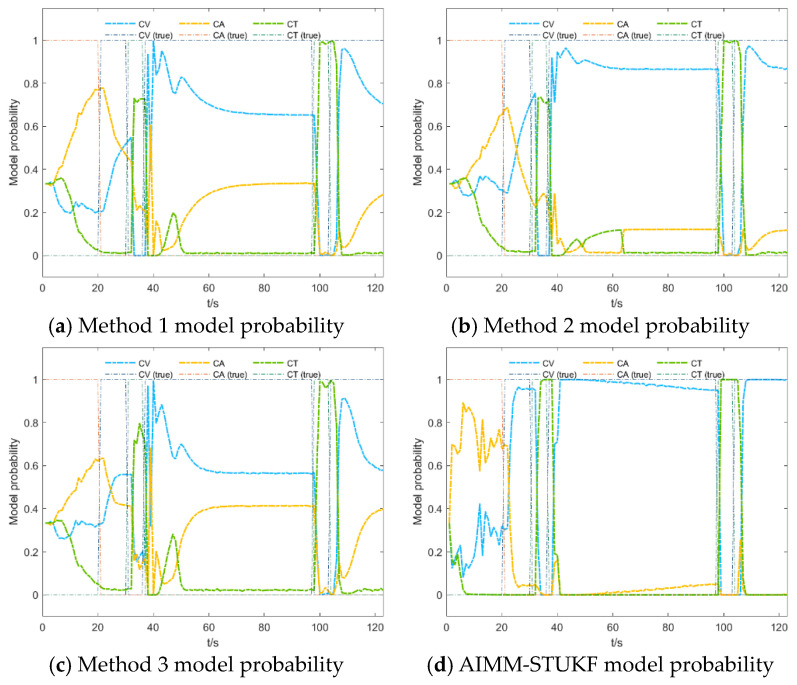
Model probability comparison of the four algorithms in Scenario 1.

**Figure 10:**
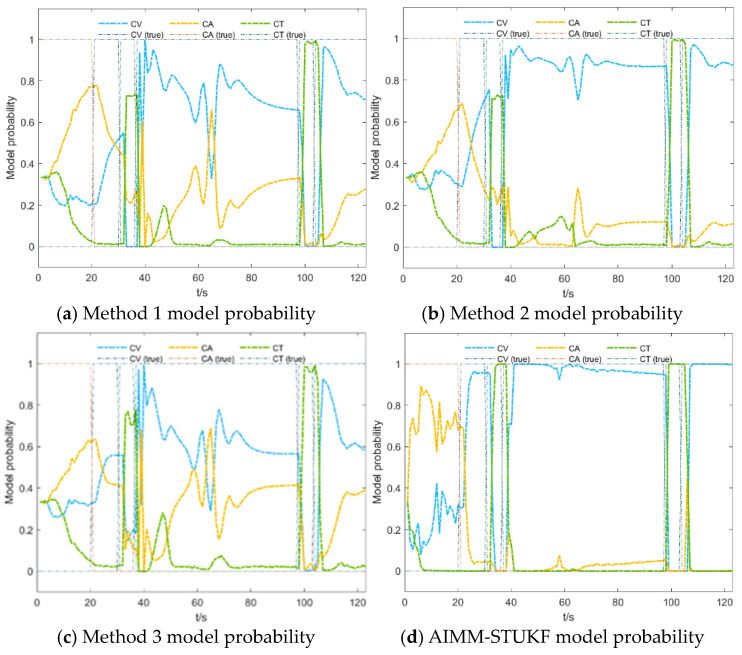
Model probability comparison of the four algorithms in Scenario 2.

Figure 9 and Figure 10 compare the model probability evolutions of the four algorithms under Scenarios 1 and 2, respectively.

(2) Analysis of results

Based on Table 1 and Table 2, the AIMM-STUKF algorithm consistently outperforms the other four methods across 100 Monte Carlo runs, both under normal driving conditions and in scenarios with abrupt disturbances. The average RMSE values for position and velocity are significantly lower, indicating substantial accuracy improvements. Moreover, the peak RMSEs of both position and velocity are markedly smaller than those of the comparative algorithms, further highlighting the clear advantage of the AIMM-STUKF approach. Quantitatively, compared with the conventional IMM–UKF, the proposed algorithm achieves pronounced performance gains. Under the normal-driving condition, the average position and velocity RMSEs are reduced by 75.697% and 58.547%, respectively, while the peak position and velocity RMSEs decrease by 87.206% and 56.483%. Under the disturbance condition, the average position and velocity RMSEs are reduced by 61.408% and 57.13%, and the corresponding peak RMSEs decrease by 53.765% and 53.860%, respectively. These results confirm that the proposed method achieves markedly higher estimation accuracy and robustness across both operational scenarios.

From Figure 5 and Figure 6, it can be observed that the proposed algorithm consistently yields lower root-mean-square errors (RMSE) in both position and velocity throughout the entire motion period, with particularly significant improvements during turning maneuvers and at the unstable transition points following turns. In the position RMSE profiles, the other three algorithms exhibit pronounced oscillations during turns and show slow recovery to steady performance after the motion-state transition, resulting in persistently elevated RMSE values, whereas the AIMM-STUKF curve remains smooth and stable. Under impulsive perturbations, the proposed algorithm also demonstrates substantially smaller peak RMSEs during disturbance intervals. A similar pattern is observed in the velocity RMSE results: in both scenarios, the proposed method yields markedly smaller RMSE peaks during turns and disturbance phases, and it restores stable performance much more rapidly following motion-state changes. It is also observed that the velocity RMSE is generally larger than the position RMSE, which can be attributed to the characteristics of the framework and the adopted motion models, where velocity components are more sensitive to model mismatch and larger inter-model differences arise during model interaction and fusion. These results indicate that the proposed algorithm exhibits superior tracking accuracy, dynamic adaptability, and disturbance rejection capability under complex motion conditions—such as turning, state transitions, and abrupt perturbations—thereby validating its applicability and advantages in airport environments involving highly maneuvering targets.

Analysis of Figure 7 and Figure 8 indicates that the proposed AIMM-STUKF algorithm exhibits significant advantages under both the normal-driving condition and the abrupt-disturbance condition. In the normal-driving scenario, the estimated trajectory remains closely aligned with the true trajectory throughout straight-line and turning motions, without exhibiting any notable deviations. Moreover, during the post-turn phase in which the motion state changes, all comparison algorithms show pronounced trajectory deviations and oscillatory behavior, whereas the proposed method maintains stable tracking and accurate localization without drift. Under the abrupt-disturbance scenario, the AIMM-STUKF algorithm effectively suppresses the impact of the disturbances in both perturbation intervals, with its estimated trajectory consistently adhering to the true path. In contrast, the other three algorithms demonstrate substantial trajectory deviations within the disturbed segments, resulting in significant discrepancies between the estimated and true trajectories.

Based on the results shown in Figure 9 and Figure 10, the proposed AIMM-STUKF algorithm achieves more accurate model-probability estimation and faster model switching. The algorithm distinctly separates the dominant and secondary motion models, with the dominant model probability approaching unity and the secondary model probability converging toward zero. When the target undergoes a change in motion state, the AIMM-STUKF enables significantly faster model transitions, completing the switching process within approximately two seconds; for example, it successfully performs two consecutive model transitions between 20 and 30 s, whereas the other algorithms exhibit noticeably slower responses and are unable to switch at a comparable rate. Under abrupt perturbations, the proposed method also demonstrates superior stability: at the perturbation instants (t = 60 s and t = 103 s), the AIMM-STUKF maintains a stable model-probability output without large fluctuations, indicating stronger robustness.

## 6. Conclusions

This study proposes an Adaptive Interacting Multiple Model–Strong Tracking Unscented Kalman Filter (AIMM-STUKF) to address the limitations of conventional IMM-UKF algorithms in airport-surface moving-target tracking. Traditional UKF-based filters are prone to divergence, rely on fixed prior transition probabilities, and fail to incorporate airport map information, resulting in degraded tracking accuracy and unreliable model-matching performance. In the filtering stage, the proposed approach adopts a standard STUKF formulation, which enhances responsiveness to abrupt motion-state changes. In addition, an exponential correction strategy based on posterior model-probability differences is introduced to adaptively update the Markov transition probability matrix in real time, thereby accelerating model-probability convergence and improving model-switching reliability. Meanwhile, airport map information is incorporated to impose constraints on the position components of the filtered state estimates, effectively reducing position deviations caused by off-route target motions. By systematically integrating real-time adaptive transition probability updating, airport map-based constraints, and STUKF-based filtering, the proposed framework improves overall tracking performance in airport environments. Simulation results demonstrate that the proposed AIMM-STUKF achieves superior tracking accuracy, more reliable model-probability estimation, and stronger disturbance rejection compared with three benchmark algorithms, indicating improved robustness and suitability for complex airport-surface operational environments.

## Figures and Tables

**Table 1:** Scenario 1 tracking results comparison.

Method	Position RMSE		Velocity RMSE	
	Average (m)	Peak (m)	Average (m/s)	Peak (m/s)
Method 1	1.148	9.098	1.597	16.467
Method 2	1.113	8.467	1.177	11.784
Method 3	0.947	8.391	1.029	12.351
AIMM-STUKF	0.279	1.164	0.662	7.166

**Table 2:** Scenario 2 tracking results comparison.

Method	Position RMSE		Velocity RMSE	
	Average (m)	Peak (m)	Average (m/s)	Peak (m/s)
Method 1	1.477	11.394	1.774	17.061
Method 2	1.423	10.898	1.302	11.784
Method 3	1.236	10.797	1.140	12.351
AIMM-STUKF	0.570	5.268	0.761	7.872

## Data Availability

The data is unavailable due to privacy and ethical restrictions.
